# Working for the environment: farmer attitudes towards sustainable farming actions in rural Wales, UK

**DOI:** 10.1007/s10668-024-04459-y

**Published:** 2024-01-31

**Authors:** Elizabeth Follett, Lorna Davis, Catherine Wilson, Jo Cable

**Affiliations:** 1 Department of Civil and Environmental Engineering, School of Engineering, University of Liverpool, Liverpool, UK; 2 Sudsplanter Ltd, Bradford on Avon, Wiltshire UK; 3 Hydro-Environmental Research Centre, School of Engineering, Cardiff University, Cardiff, Wales UK; 4 School of Biosciences, Cardiff University, Cardiff, Wales UK

**Keywords:** Sustainable farming, Environmental land management, Wales, Public goods, Land sparing

## Abstract

Recognition of land management impacts on water quality and flooding, and climate change-induced increases in storm intensity and flood risk, have led to interest in farmer provision of ecosystem services alongside food production. However, pathways for practical design and funding of agroecological interventions are less well understood. Effective design and implementation of sustainable farming initiatives have been linked to human-centred aspects including stakeholder engagement and provision of social and economic co-benefits. To obtain information on Welsh farmer perspectives on sustainable farming actions and aid development of agroecological policy and design guidance, Welsh farmer perspectives on sustainable farming were obtained through discussion, online polls, and questionnaires. Participant-identified barriers to action included incorporation of return on initial time and cost investment in long-term farm budgets, occurrence of extreme weather events, and tenanted land. Decision-making processes were rooted in community discussion to balance perceived needs of the land and farm business, with communication preferences expressed for bilingual farm advice provision and support of farmer-to-farmer knowledge transfer pathways. In addition to responding to research questions, participants identified interdependent components of economic, social, cultural, and environmental sustainability necessary to achieve positive environmental outcomes, and expressed environmentally oriented farming identities linked to environmental guardianship and caretaking. Design of tree-planting schemes was discussed as an example of this interlinkage, with positive attitudes expressed for land sharing at small spatial scales, but not at the whole-farm scale.

## Introduction

Farmers play a crucial role in sustaining ecosystem services of clean water, air, and habitat provision, as well as food production (Hewett et al., 2020; O’Connell et al., 2007). Farmers manage 85% of Wales’ land area (Wiseall, 2018), maintaining and enhancing the natural environment for aesthetic enjoyment and well-being of the wider population. Their provision of ecosystem services can range from habitat creation, due to implementation of riparian woodland, buffer strips and vegetated ditches and swales, and management of water storage by infiltration on farmland and temporary water storage in on-farm swales and ponds, to reducing flood impacts and carbon capture (Hewett et al., 2020; O’Connell et al., 2007). The benefits of agroecological schemes aiming to increase provision of ecosystem services have gained particular relevance as climate change is expected to increase summer storm intensity and temperature, increase sediment runoff and decrease water quality (IPCC, 2014; Watts and Anderson, 2016). In addition, intensification of UK agriculture following the Second World War has increased soil compaction, runoff from bare soil, and river channelisation, increasing the potential for sediment loss and decreased downstream water quality (Boardman et al., 2003; O’Connell et al., 2007). The importance of farming practices in protecting ecosystem services, including downstream water quality (Belmont et al., 2011; Carroll et al., 2006; McIntyre et al., 2014; Schottler et al., 2013), has led to widespread international interest in recognising and rewarding agricultural land management actions expected to lead to positive environmental outcomes (Burton et al., 2008, NFU Cymru, 2018, Welsh Government, 2019a; OECD, 2020; Reilly & Mercier, 2021; Cusworth & Dodsworth, 2021). Internationally and in Wales, farmers have called for technical advice and funding to aid adaptation to climate change-induced stressors and provide compensation for practices that increase provision of ecosystem services (Case, 2021; Graddy-Lovelace, 2020; Hyland et al., 2016; Kummer, 2021).

The role of farmers in provision and maintenance of ecosystem services has been recognised (Belmont et al., 2011; Hyland et al., 2016; O'Connell et al., 2007), Kummer, 2021). Early studies identified tension between environmentally sensitive farming practices and productivism-oriented goals towards high yields and intensification (Burton, 2004; Burton & Paragahawewa, 2011; Burton et al., 2008). However, more recent investigations have observed an integration of farmer identities with ecosystem service provision following increasing awareness of environmental issues, higher prices paid for organic produce, and introduction of subsidisation schemes for provision of ecosystem services via sustainable farming actions, including in Wales (Cusworth & Dodsworth, 2021; Hyland et al., 2016; Sutherland & Darnhover, 2012). Bourdieu’s social theory, in which economic, social, and cultural capital are required to generate symbolic capital, has been used as a framework to explain the integration of positive views of environmentally sensitive farming practices with farmer identities and practices (Bourdieu, 1986; Cusworth & Dodsworth, 2021; Hilgers & Mangez, 2015). In this framework, subsidisation for sustainable farming actions that increase provision of ecosystem services (economic capital), recognition and awards via participation in agrienvironmental schemes or organic practices (cultural capital) and increasing awareness of environmental issues among a farmer’s network of contacts (social capital) facilitated implementation of sustainable farming actions in England (Cusworth & Dodsworth, 2021). In particular, land-sparing strategies, in which less-productive farmland is set aside for public goods provision, was seen to integrate well with productivist identities. Practices that allow farmers greater autonomy, harnessing farmer expertise in optimisation, efficiency, and maximisation of outputs in designing and implementing food and public goods provision were recommended (Cusworth & Dodsworth, 2021).

Recognition of the complex social, economic, and political aspects related to farming practice (Burnett, 2023) has led to calls for development of designs that support local social, economic, and political needs, termed people-centred nature-based solutions (Fleischman et al., 2020). The concept of such solutions echoes broader international standards for human-centred design targeting interactive systems. The International Standards Organisation requires that human-centred approaches base design on six principles including (1) an explicit understanding of users, tasks, and environments, (2) incorporation of users throughout design and development, (3) that design is driven and refined by user-centred evaluation, (4) that the process is iterative, (5) the design addresses the whole user experience, and (6) that the design team includes multidisciplinary skills and perspectives (ISO, 2019). Stakeholder involvement in codesign harnesses available expertise in optimal placement and design of interventions to maximise benefit to the farm business (Cusworth & Dodsworth, 2021). Further, focus on the inner drivers for farmers’ choices and perspectives towards agroecological schemes has been advocated to enable sustained, transformational changes based on investigation of farmer pathways to change in the Netherlands (Bakker et al., 2023). More broadly, successful implementation of nature-based solutions for natural flood management has been linked to human-centred aspects including stakeholder engagement and the provision of social and economic co-benefits. A catchment-wide approach including stakeholder involvement in knowledge co-production, co-creation, and intervention design has been advocated for successful implementation of catchment systems engineering (Hewett et al., 2020).

Additional challenges for intervention design and implementation raised by farming in resource-poor regions have been highlighted historically (Pretty, 1991) and in contemporary examination of agroecological schemes (Fraser et al., 2014). In Wales, land use is dominated by grassland pasture due to high prevalence of upland, mountainous regions, and wet climate, with 61% of Welsh farmland classed as a Less Favourable Area (LFA) for agriculture. Examination of conversion from conventional to organic farming methods within LFAs in England and Wales led Fraser et al. (2014) to observe that limited increases in habitat diversity were linked to constrained management options due to physical conditions of the LFA, leading to calls for additional evidence related to farming and agroecological management in such regions.

Our study objective was to obtain information on Welsh farmer perspectives on sustainable farming actions, to aid development of agroecological policy and design guidance by deepening understanding of user approaches and perspectives. Welsh farmer perspectives on design and implementation of sustainable farming actions were investigated through an engagement project centred on four research questions: (1) What are the main barriers to implementation of sustainable farming actions by Welsh farmers?, (2) What is the decision-making process of Welsh farmers towards sustainable farming actions?, (3) How should information on sustainable farming be communicated to Welsh farmers?, (4) What suggestions do farmers have for the future of sustainable farming in Wales? In addition to directly addressing project research questions, participants built on the term “sustainable farming” to highlight broader interlinkage between environmental sustainability and social, economic, and cultural aspects of sustainability. The identification of an emergent theoretical framework from collected data could be viewed as approaching questions from a grounded theory perspective, in which theoretical concepts emerge from data collected during the research process (Corbin & Strauss, 2015; Glaser & Strauss, 1967; Vollstedt & Rezat, 2019). Project data were read and coded in between the initial and final workshops, allowing verification and revision of emerging concepts.

## Methods

**Fig. 1:**
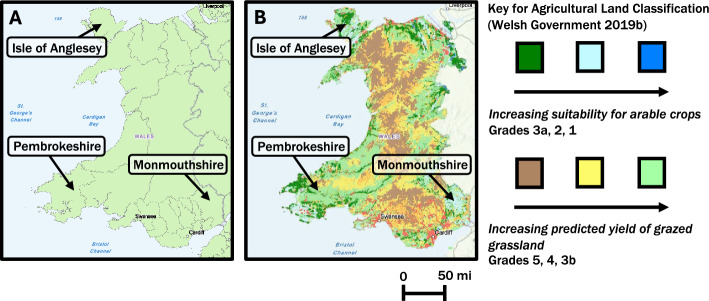
(**A**) Map of survey areas in Wales, UK: Isle of Anglesey, Monmouthshire, and Pembrokeshire counties indicated by solid black arrows. County boundaries grey solid lines (UK Office of National Statistics 2018). Border with England denoted with solid dark grey line. (**B**) Classification of agricultural land (Welsh Government, 2019b)

Project delivery targeted three counties across Wales (Monmouthshire, Pembrokeshire, and Anglesey; Fig. 1). Pembrokeshire contained a higher prevalence of arable cropland (Welsh Government, 2017), and Anglesey and Pembrokeshire a higher percentage of Welsh speakers (67%, 32%; Monmouthshire 18.8%; Welsh Government Office for National Statistics, 2022). A project summary was provided to farmers’ union county representatives along with a supporting e-mail in Welsh and English. Representatives advertised workshops via email and orally at county meetings. Phone calls were made to at least 25 farmers per county known to have an interest in sustainable farming. To reach a broad audience of potential participants (6000 member farmers) and provide a transparent overview of project goals to the community, a project outline was published in the NFU Cymru magazine (July 2021).

Online workshops (July 2021) and in-person visits (August, September 2021) presented information on sustainable farming actions leading to open-ended discussion around project research questions, supported by online polls or paper questionnaires depending on meeting setting. Content focused on water quality measurement methods and sustainable farming actions applicable to grazed and arable land, including buffer strips, sediment traps, sediment barriers, vegetated ditches and swales, and preventing surface runoff from yard areas. An initial set of three meetings targeted individual counties (July 2021; 4, 3, and 3 participants). Anonymised transcripts of discussion were collated during the meeting, and anonymous poll responses were visible to participants and downloaded. In-person meetings were hosted on participant farms in Pembrokeshire (August 2021; 9 participants) and Anglesey (September 2021; 11 participants), with anonymous paper questionnaires replacing online polls. On-farm walkover visits were offered to all participants, with two participants accepting. Short information sheets were presented at the Pembrokeshire Fair (20 visitors, August 2021). Following in-person meetings, an interview was conducted with the project member leading the visit, recorded via written summary (September 2021). A summary online workshop held for all counties (November 2021; 5 participants) described the coding procedure and presented initial results including a summary of Table 2, draft Fig. 3, illustrated infographic, and technical drawings for a related art piece to participants for verification and revision. In total, the initial Workshop 1 online series had 10 participants, 20 participants attended in-person meetings, and a booth at the Pembrokeshire fair received 20 visitors, often short interactions. The final Workshop 2 had 5 participants, including county officers for each of the 3 target counties, who represented broader county perspectives.

### Qualitative data analysis and sampling procedure

Themes arising from meeting transcripts, post-visit interviews, and feedback forms were identified using a manual coding and categorisation process (Burnard, 2004; Burnard et al., 2008). Five extrinsic codes were identified from the four project research questions and goal to collect feedback on project outputs: “barriers to implementation,” “decision-making process,” “communication preferences,” “future of farming,” and “feedback on project outputs.” During the initial reading, two further intrinsic codes were identified: “working for the environment” and “aspects of sustainability.” Project data were then re-read and each sentence assigned one of the seven codes (Table 1). Initial results were presented to participants for verification (November 2021). Notes from this meeting were read and coded using the structure applied to earlier data.

**Table 1:** Codes used in qualitative data analysis, origin from project research questions or emergent from data, definition, and example quote related to code

Code	Origin	Summary	Example
Barriers to implementation	Extrinsic	Related to RQ1: “What are the main barriers to implementation of sustainable farming actions by Welsh farmers?”	“Why would I invest £15k in a pond, cost to put it in exceeds the value they think it will deliver”
Decision-making process	Extrinsic	Related to RQ2: “What is the decision-making process of Welsh farmers towards sustainable farming actions?”	“I’ve got a photo of clear water coming out of our land drain and a maize field across the road with sediment, it shows how much filtering through the soil matters.”
Communication preferences	Extrinsic	Related to RQ3: “How should information on sustainable farming actions be communicated to Welsh farmers?”	“It should be two-way, having these conversations. I’m happy to get my voice heard.”
Future of farming	Extrinsic	Related to RQ4: “What suggestions do farmers have for the future of sustainable farming in Wales?”	“Some farmers are already putting in cover crops for example as part of their retailer contract and rewarded for doing so. This will only increase as pressure builds on the climate change debate.”
Feedback on project outputs	Extrinsic	Feedback on project engagement outputs (sampling toolkit, information sheets)	“Haven’t read the info sheets. Too long and too much text.”
Working for the environment	Intrinsic	Expression of identity related to environmental objectives	“I want others to understand that we are working for the environment”
Aspects of sustainability	Intrinsic	Definition and interrelationship between aspects of sustainability raised by participants	“Environmental advances only come when people want to do them, there’s value back to business, and they’ve got a real use. What is the wider benefit?”

Project data was read after each initial workshop and reviewed in order to improve workshop delivery. For example, several participants connected to workshops using phones in farm fields and were not able to respond to online polls, so subsequent workshops acknowledged this difficulty and offered opportunity for responses to be given verbally. Project data was read after each meeting, and re-read and coded in October and early November 2021, before Workshop 2. A draft summary of response to each research question, identification and summary of emergent project themes, and draft table of barriers to action and figure showing framework for interrelated aspects of sustainability) were created following the initial coding process. The figure was revised following discussion with participants in Workshop 2. Questions for discussion in Workshop 2 included the original four project research questions, as in the initial project meetings, and questions on emerging project themes of “working for the environment” and “aspects of sustainability” (Table 1).

**Table 2:** Main barriers to implementation of sustainable farming actions identified by project participants, with identified practical impacts

Barrier	Impact
Long-term economic valuation	Justification of time and cost to implement action in business plan Lack of access to bank loan without clear return on investment Recognition of action by farm assurance schemes
Extreme weather events	Extreme flooding may overwhelm capacity or occur at inopportune time in tillage cycle, causing sediment and nutrient loss despite action taken Fines and negative public perception linked to extreme event may persist despite action Actions perceived as not working for intended purpose, even if effective at lower to moderate flow levels Increased frequency of extreme rainfall and drought stresses farm business, reducing available time and money to implement actions
Tenanted land	Reduced decision-making power and long-term investment Associated with reduced social stability and economic prosperity

**Fig. 2:**
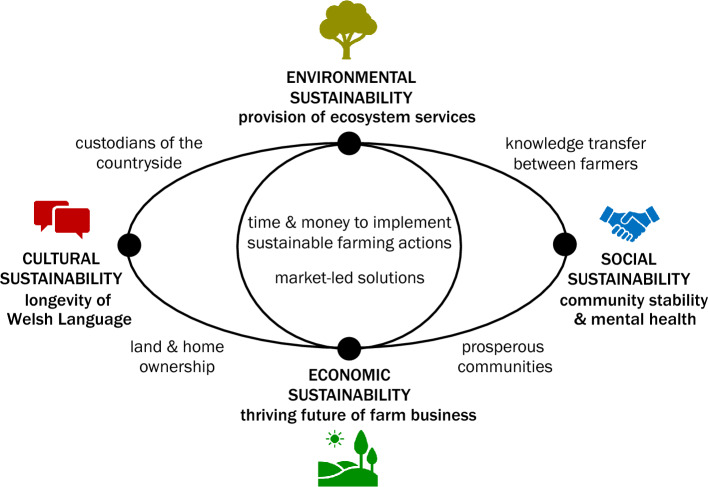
Definitions and spatial relationship (black lines) between environmental, economic, social, and cultural aspects of sustainability identified by project participants (black solid circles). The centrality and interdependence of economic and environmental sustainability were emphasised during verification by participants in Workshop 2

### Method limitations

The low sample size and voluntary participation in project meetings likely introduced self-selection bias. To address this issue, in-person workshops included a booth at the Pembrokeshire Fair targeting a wide audience, and planned 1:1 farm walkover visits were modified to hold in-person workshops on host farms to increase participant numbers. In addition, farmers union county representatives from Anglesey, Pembrokeshire, and Monmouthshire, who represented broader community opinions, were present at the final summary workshop to assist in verification and revision of project results. Where possible, we have contextualised our results in terms of 232 responses to the Welsh Government “Sustainable Farming and Our Land” consultation (O’Prey et al., 2021), and analysis of 286 Welsh beef/sheep farmers’ perspectives on climate change (Hyland et al., 2016) obtained through representative convenience sampling (Luschei et al., 2009).

## Results

Participants responded to the four project research questions, highlighting barriers to action, farmer decision-making process for sustainable farming actions, communication preferences, and suggestions for the future of sustainable farming in Wales (Sects. 3.1–3.4). In addition, emerging project themes including environmentally oriented farmer identities and identification of interlinked aspects of sustainability are presented following the four original research questions (Sect. 3.5).

### Barriers to action

Participants identified key barriers to implementation of sustainable farming actions including time and cost, extreme weather events, and prevalence of tenanted land (Table 2). Participants felt constrained by time and money in farm business budgets, and by lack of available long-term financial valuation for sustainable farming actions:

Although farmers are keen to do these works they are concerned about loss of land and lack of reward.

All this costs money. If we had more money, we could afford to do more things.

Lack of financial valuation presented barriers to incorporation of sustainable farming actions in farm budgets and obtaining funds on loan:

Just talking to the bank about silage pits, they say this will cost between £400-500k. There’s no money [to loan] because there’s no return on it.

Similarly, participants expressed concern that investment in sustainable farming actions would not be incorporated within farm assurance schemes, such as the Red Tractor, due to the lack of explicit financial valuation for associated provision of ecosystem services. For example, some participants in on-site meetings identified wet areas containing trees as *“non-productive land”* because ecosystem services provided by such areas did not form part of a long-term business plan and associated budget. In some cases, however, participants identified cost as a positive factor contributing to the adoption of sustainable farming actions, with an increase in fertiliser cost (Autumn 2021; BBC Scotland 29 September 2021) seen as a positive factor contributing to adoption of on-farm composting and nutrient retention measures to reduce the need to purchase fertiliser from outside sources.

Participant-identified time and cost barriers echoed attitudes of Welsh farmers exhibiting positive attitudes towards productivism (49%, Hyland et al., 2016) and farmer attitudes towards sustainable farming actions internationally (Herzon & Mikk, 2007; Holstead & Kenyon, 2011; Kik et al., 2021). Time and cost were viewed as greater barriers for tenant farmers, with some participants expressing an inability to implement significant alterations to rented land due to additional time needed for discussion and lack of long-term investment potential.

Weather patterns emerged as a significant additional barrier, both directly due to limited availability of dry, sunny weather conditions facilitating installation of sediment traps and ponds:

Can’t put it in if too wet, can’t do it if the weather won’t allow,

and indirectly due to occurrence of extreme weather events. Heavy rain in mid-May 2021 was brought up as an example of an extreme condition during which sediment loss would have occurred despite existing sustainable tillage practices:

We had 8-10 inches of rain in May. How can anyone manage that?

Participants expressed concern that occurrence of an extreme rainfall event at an inopportune time, for example just after tillage, could result in excess off-farm sediment and nutrient discharge despite implementation of sustainable farming actions. This raised concern that implementation would not reduce negative public perception or farmer time and financial burden.

### Decision-making process

In evaluating whether or not to implement a sustainable farming action, discussion with family, business partners, and other farmers emerged as a key decision-making step in choosing whether or not to implement a specific action and develop intervention design (*“a chat always comes first”*). Codesign of interventions and policy was viewed as a pathway to improve practical implementation and positive environmental outcomes.

Through discussion, participants attempted to balance needs of the land and farm business. Participants demonstrated a practical knowledge of the response of their land to storm conditions, and had considered placement and design of sustainable farming actions in order to make the best use of the available land while optimising the needs of the land and farm business. For example, the observed hydrologic response of land to storms was considered in farmer self-design of tree-planting schemes, including identification of wet areas and locations with frequent overland flow. Trees were described as occurring on the margins of farm fields and in wetter areas unsuitable for heavy grazing or arable crops. Self-designed tree-planting schemes in some cases were viewed as primarily generating ecosystem services (windbreak, capture of water, buffer for overland flow) and in others were considered as a long-term investment crop. Participants expressed positive views of trees as a wood products cash crop, windbreak for adjacent fields, and source of natural beauty. In some cases, participants felt restricted by policy. For example, in some cases participants gave an example of an implemented sustainable farming action (tree planting and a vegetated swale) that they viewed as fitting the overall broad goals of a sustainable farming scheme, but for which reimbursement was not possible due to an adapted intervention design that fell slightly outside of the scheme regulations. In addition, the negative impact of the design of narrow fenced buffer strips, which resulted in invasive bramble growth and reduced biodiversity compared to an occasionally grazed or mowed strip, was brought up by participants:

Experience of streamside corridors have so far been disappointing as the lack of grazing means that it becomes inaccessible for everyone and everything and actually reduces biodiversity.

Participants expressed a willingness to engage in future two-way conversation and codesign of interventions and policy:

There’s a lack of being able to design for your farm. It’s too prescriptive.

Should be two-way, designing toolkit and policy—Brilliant! Codesign policy is how it should be. That’s why I’m doing it. I want my voice to be heard.

Participation in policy and intervention codesign was seen as a pathway to improve correspondence with practical implementation and positive environmental outcomes for sustainable farming actions.

### Communication preferences

Discussion with family, business partners, and other farmers and reliance on the local farming community emerged as a key step in both decision-making and knowledge transfer. Participants identified discussion with other farmers as a preferred communication pathway:

Community is very strong—this is very important in doing business in terms of knowledge transfer between farmers.

Discussion amongst other farmers in knowledge sharing groups is really important and I value this a lot. This is different to groups in which people want to sell you something or get you to do something. I value discussion amongst farmers.

Participants commented positively on the advantages of online meetings, especially in reducing fuel costs and connecting regions as far afield as the UK and Australia.

Zoom is a massive step forward. From NFU-engage with anybody in the world.

The limitations of *“poor broadband [availability] and poor digital connection”* were brought up as a disadvantage in some rural areas. Despite the benefits of online meetings, participants felt that in-person meetings had some additional benefits not possible online:

It’s the way forward, but you miss that personal discussion after the meeting.

Recipients of 1:1 farm walkover visits highlighted the value of in-person, on-farm discussion in identifying practical application and design of sustainable farming actions. Following consultation with participants, project catchment-wide (Workshop 1, July 2021) and cross-catchment (Workshop 2, November 2021) meetings were held both to meet COVID-related restrictions (July 2021) and to address practical travel time and cost limitations in conducting a group workshop for all catchments (November 2021), which were located across Wales (Fig. 1). In addition to learning from community members, participants expressed appreciation for the two-way conversational nature of project workshops and co-production of project outputs.

### Suggestions for the future of sustainable farming in Wales

Despite identified barriers, participants were optimistic about the future of farming in Wales (*“We love the industry, that’s why we do it”*) and expressed a willingness to be *“part of the solution”* (*“Lots could be done with a little bit of joined up thinking, really”*)*.* Participants expressed a desire for future two-way conversations and codesign of agricultural policy and sustainable farming actions. In addition, participants suggested mechanisms for economic valuation of ecosystem services provided by sustainable farming actions, in the hope that this would reduce identified time and cost barriers to implementation and allow incorporation of investment in sustainable farming actions into farm business plans. Suggestions included payments for *“ecosystem services as a public good”* such as water filtration and potential flood alleviation, as well as management of *“highway runoff”* entering farm fields.

Participants expressed a strong desire for market-led solutions driven by positive public perception of sustainably produced food. Participants noted increasing interest in sustainable farming actions from retailers as positive public perceptions of sustainable farming trickled upwards to farmers:

Some farmers are already putting in cover crops for example as part of their retailer contract and rewarded for doing so. This will only increase as pressure builds on the climate change debate.

Farmer-owned cooperatives were advocated as a social tool to build pathways for knowledge transfer between cooperative members and *“support changes within that system.”* Here, long-term availability of knowledge transfer pathways and amount of time and money (Table 2) to implement sustainable farming actions is ultimately driven by any profits generated by the cooperative group. However, some participants raised caution over the cooperative model as a pathway to long-term success (*“There’s a big history of failures. They need to be run well”*). A combination of strategies with an emphasis on market-led solutions was advocated as a practical way forward.

Participants in Pembrokeshire and Anglesey emphasised the cultural and practical importance of the Welsh Language, including that county farmers union meetings were usually conducted in Welsh.

### Emerging project themes

Participants demonstrated a shared feeling of pride in farmers’ traditional role as “*custodians of the land*.” Hedgerow trimming and coppicing of tree stands were highlighted as examples of traditional environmental guardianship:

I want others to understand that we are working for the environment.

Who would trim the hedgerows if the farmers didn’t do it? What would the land look like? It’s a beautiful landscape.

We all want clean water and love the environment, but us farmers can’t produce for what it costs.

Participants felt “*under appreciated, misunderstood, undervalued*” by the perceived depiction of farmers as uncaring or polluters. In contrast, participants felt that existing barriers prevented farmers from wider implementation of sustainable farming actions.

In addition, participants built on the term “sustainable farming” to identify multiple aspects of sustainability including environmental, economic, social, and cultural sustainability (Fig. 2, solid black circles) and the relationship between these aspects, depicted by lines. Social, cultural, and economic sustainability were identified as interlinked components necessary to achieve environmental sustainability:

There are different types or components to sustainability: social, economic, environmental

There’s a cultural aspect, we’re Welsh speaking areas; rural, farming communities are guardians of the Welsh Language, so there’s a cultural aspect of sustainability around that as well.

Participants identified economic sustainability as a necessary condition for social and environmental sustainability. Sustainability was seen as “*embedded in stability,”* and the four identified aspects were described as *“intertwined in a lot of ways.”* Economic success was seen as contributing to business longevity and farm ownership, in turn benefitting social sustainability through positive contributions to the welfare of the local community, including long-term development of community social links. A healthy local community was seen as providing two-way benefits to a thriving local economy. Participants associated social stability with cultural stability and Welsh Language longevity, as spoken Welsh could continue to be passed down to the next generation.

News of the purchase of farmland in Carmarthenshire, Wales, as part of a tree-planting scheme for carbon capture by the London-based investment company Foresight Group in Summer 2021 (Garside & Wyn, 2021) was brought up in multiple meetings as an example of an effort to achieve environmental sustainability to the detriment of local social, cultural, and economic sustainability. Purchase of farmland for tree planting by an external group was seen as generating value primarily outside the local community, reducing local economic sustainability. Ownership of local land by a corporation was considered as reducing locally available farmland and housing, reducing social sustainability. A lack of local employment and housing options was perceived to be connected to movement of local young people to regions with a weaker or no Welsh cultural and linguistic identity, reducing cultural sustainability. While the tree plantation was viewed as generating a medium-term (decades-long) environmental benefit due to carbon capture by growing trees, participants expressed concern about the net longer-term carbon offset benefit of tree planting, which in part depended on the tree end use. Concern for the *“wider benefit”* extended to a range of sustainable farming actions, with participants advocating a strong desire for involvement that made a positive practical contribution to environmental health and climate change.

## Discussion

Welsh farmers highlighted interlinked, economic, social, cultural, and environmental factors needed to achieve rural sustainability. To contextualise our results, we first consider the emerging interlinked themes of sustainability and design of tree-planting schemes raised by participants, and then the barriers and solutions to more sustainable farming.

### Emerging theme 1: interlinked aspects of sustainability

The farmer-identified framework of interrelated economic, environmental, social, and cultural aspects of sustainability (Fig. 2) resembles Bourdieu’s social theory, in which economic, social, and cultural capital are required to generate symbolic capital (Bourdieu, 1986; Cudsworth & Dodsworth, 2021; Hilgers & Mangez, 2015). Increasing provision of economic, social, and cultural capital via subsidisation schemes and increasing awareness of environmental issues has been used to explain integration of English farmer identities with provision of public goods via sustainable farming actions in a Bourdieuian framework (Cusworth & Dodsworth, 2021). Similarly, in the framework generated by study participants (Fig. 2), sufficient support for economic, social, and cultural aspects was required to achieve a successful environmental outcome. However, participant definitions of economic, social, and cultural aspects expressed in this study differed from classic definitions (Bourdieu, 1986; Cusworth & Dodsworth, 2021). Participants emphasised the interconnectedness of economic, social, cultural, and environmental components of sustainability and a long-term outlook. For example, economic success was seen as not simply availability of funds, but contributing to business longevity and farm ownership. Economic sustainability was described as both the incorporation of sustainable farming actions into long-term business plans to realise return on initial investment, and long-term economic confidence that the farm business would be profitable and survive to be passed down to the next generation. The presence of economic sustainability was seen as contributing positively to social sustainability, improving mental health and community welfare. Participant linkage of social sustainability to long-term community stability emphasises interconnectedness and the long-term beyond classic Bourdieu definitions of social capital as a network of contacts. In turn, a stable and well-supported community was viewed as conducive to a thriving local economy. Participants associated economic and social stability with a separate and location-specific component of cultural stability, which participants linked to longevity of Welsh culture and language. Spoken Welsh was expressed as being transferred through stable intergenerational family networks and serving as the primary language of farmers’ union county meetings in Pembrokeshire and Anglesey.

In addition to identifying interlinkages between economic, social, and cultural aspects and positive environmental outcomes, participants viewed environmental contributions as linked to broader cultural identity and impact, taking pride in their longstanding caretaking role as *“guardians of the countryside”* and *“working for the environment.”* For example, hedgerow trimming and coppicing were highlighted as examples of traditional environmental guardianship, echoing motivations expressed by 51% of respondent farmers exhibiting high levels of environmental responsibility (Hyland et al., 2016). However, due to the small sample size and self-selection bias, project participant views were likely more representative of farmers with higher levels of knowledge and engagement with sustainable farming actions rather than the community as a whole. An investigation of English farmers also observed generally positive attitudes towards delivery of diverse farming objectives including ecosystem services and food production, suggesting that widespread recognition of the expanding economic market for farmer-provided services had reduced previous negative associations with untidiness and non-productivity (Burton et al., 2008; Cudsworth & Dodsworth, 2021).

### Emerging theme 2: perceptions of tree-planting scheme design

**Fig. 3:**
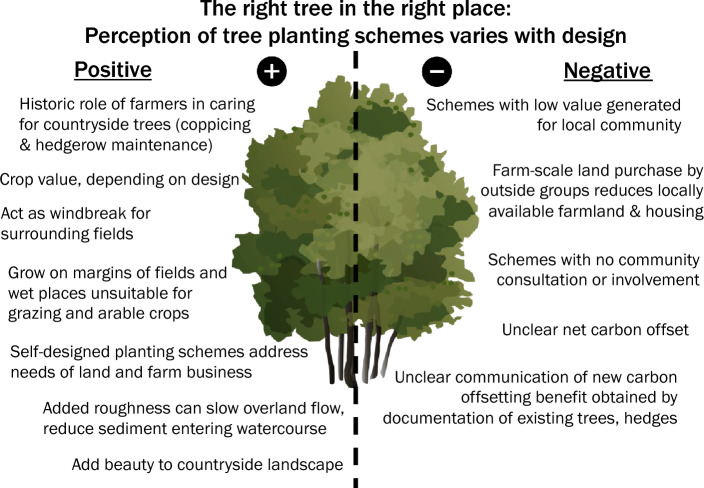
Positive and negative attributes of tree planting identified by project participants

Design of tree-planting schemes was highlighted as an example of the participant-generated framework (Fig. 2), with a successful environmental outcome requiring consideration of interlinked economic, social, and cultural aspects over the long term. Participants favoured support of pre-existing living trees and self-design of tree-planting schemes that complemented their existing livelihood (Fig. 3), with participants expressing positive attitudes towards existing countryside trees and farmers’ role in caring for historic tree stands, in some cases hundreds of years old, through coppicing and hedgerow maintenance. Existing countryside trees and farmer self-designed tree-planting schemes were described as occurring on the margins of farm fields and in wetter areas unsuitable for heavy grazing or arable crops. Positive participant attitudes towards tree planting in areas generally viewed as unsuitable for other agricultural use is consistent with previous observations of farmer preference for “land sparing,” in which part of a field, often marginal land, is set aside for generation of ecosystem services (Cudsworth & Dodsworth, 2021).

Although participants expressed positive attitudes towards land sparing at small spatial scales, strong negative viewpoints were expressed for land sparing at the larger whole-farm scale (Fig. 3), linked to the recent purchase of Carmarthenshire farmland for use as tree plantations for carbon offsetting by the London-based Foresight Group (Garside & Wyn, 2021). Alteration of ownership and management of farmland at this scale was linked to loss of local housing and livelihoods, due to the farm-scale of the purchase of land by an outside group, instead of individual farmers residing in the community, and associated low expectations for local employment. In this type of scheme design, whole-farm scale land purchase was seen as negatively affecting the local economy and negatively impacting local social and cultural ties, reducing confidence that young people would be able to remain in the local community. Participants worried that due to the strong market for carbon credits, such purchases could become commonplace, eliminating rural farming communities.

Participant concerns echoed recent results examining the complexities of tree-planting design internationally (Coleman et al., 2021; Fleischman et al., 2020). An analysis of 50 years’ investment in Indian tree-planting schemes (Coleman et al., 2021) revealed no net increase in canopy cover. Because targeted areas were already under use as farmland, most planting occurred in marginal areas with tree cover already present. In response to concerns generated by analysis of existing tree-planting schemes, development of people-centred nature-based solutions that support local social, economic, and political needs has been advocated (Fleischman et al., 2020).

### Barriers to, and recommendations for, sustainable farming in Wales

Participants expressed positive views of sustainable farming actions but were limited from further implementation by time and cost barriers, underscoring the complex physical and social nature of implementing nature-based solutions for natural flood management and sustainable farming (Wingfield et al., 2021) and advantages of incorporating human-centred aspects into design of nature-based solutions (Fleischman et al., 2020). The participant-generated framework of interlinked support for economic, social, and cultural aspects in order to achieve successful environmental outcomes (Fig. 2) can be used as a framework to examine farmer barriers to action and suggested pathways forward in Wales.

Participant-identified economic barriers of time and cost, as well as the central link between economic and environmental sustainability, echoed attitudes of farmers and value chain participants in a study of soil management in the Netherlands, where economics and income were prioritised as key criteria for sustainable soil management (Kik et al., 2021). Scottish farmers expressed major concerns about the effect of natural flood management measures on business viability, with 58% of 193 survey respondents indicating that more funding would encourage them to implement such measures (Holstead & Kenyon, 2011). Similarly, Estonian farmers highlighted concerns for environmentally beneficial land management measures that affected farm productivity, with farmers who viewed environmental issues as more important being more likely to implement such measures (Herzon & Mikk, 2007). To reduce economic barriers, participants emphasised market-led methods but suggested a combination of mechanisms including valuation of practices that increase ecosystem services in retailer contracts, farm cooperative business models, government subsidy programs, and development of explicit financial valuation for ecosystem services linked to sustainable farming actions, enabling inclusion in lending models and farm assurance schemes. Similarly, responses to recent consultation advocated the positive benefits of a mix of market and government support in both provision of public goods and food production, with government subsidies acting as a protection from market fluctuations (O’Prey et al., 2021; Welsh Government, 2020b). While respondents to a Welsh Government consultation on agroecological policy (O’Prey et al., 2021) expressed a generally positive attitude towards sustainable farming actions, respondents also raised the need for regulatory mechanisms to enforce compliance with environmental regulations including fines and criminal prosecution for serious offences. A proportional regulatory scheme was advocated to create a non-confrontational regulatory environment where possible (O’Prey et al., 2021).

Participant preferences for communication pathways that support local social and cultural links, such as community discussion and knowledge transfer among farmers, were consistent with positive outcomes of farmer-to-farmer training models (Kansaga et al., 2021). A targeted one-to-one advice model was recently expanded in England to cover all catchments as part of increased investment (£30 M/annum) for the Catchment Sensitive Farming programme (Defra Press Office, 2 August 2021). Participants in this study who elected to receive an on-farm walkover visit felt that in-person, on-farm discussion provided practical value in identifying options for implementation of sustainable farming actions. In a recent public consultation period (“Agriculture (Wales) White Paper,” Welsh Government, 2020a, 2021), respondents emphasised the importance of provision of farm advisory services (Farming Connect) and clear communication of targeted advice and guidance in improving farmer knowledge and implementation of new regulations. The necessity of considering overall cost and practicality of advice provision was emphasised (O’Prey et al., 2021). Efforts to develop and refine digital tools to initially identify and target farm advice, such as the EU Horizon 2020 FAIRshare project (Kelly, 2019) could assist in broadening targeted advice provision while maintaining or reducing overall costs. In addition to expressing a preference for community discussion and farmer-to-farmer knowledge transfer, project participants in Pembrokeshire and Anglesey emphasised the cultural and practical importance of the Welsh Language, including that county farm union meetings were usually conducted in Welsh. Welsh-speaking advisors may be of particular interest in these and other areas where Welsh is commonly used. Use of spoken Welsh was also highlighted in consultation response (O’Prey et al., 2021).

Participants emphasised interconnectedness, a long-term outlook, and two-way conversation in navigating barriers towards implementation of sustainable farming actions and in identifying pathways forward. Existing Welsh policy initiatives targeting interconnected social, cultural, and economic aspects (Fig. 2), such as the Well-being of Future Generations (Wales) Act 2015 and Farming Connect (Welsh Government, 2015, 2023), could serve as a basis for future policy support. Welsh-speaking farm advisors, identified and supported in a broader sample of consultation respondents (O’Prey et al., 2021), could provide additional support for cultural sustainability in developing environmentally sustainable farming practice. To reduce costs, support for farmer-to-farmer knowledge transfer, a key communication preference, and digital tools could be used to broaden targeted advice provision. While the small sample size and voluntary participation of study participants likely introduced a self-selection bias towards participants with positive environmental viewpoints, participants communicated a strong personal and cultural identity of environmental guardianship, expressing pride in farmers’ longstanding role as caretakers of the countryside (Sect. 3.5) and a willingness to engage in two-way conversation and policy codesign processes (Sect. 3.2). A broader sample of Welsh farmer survey responses indicated that 51% of respondents exhibited high levels of environmental responsibility (Hyland et al., 2016). Development pathways for policy and design guidance that build on positive environmental viewpoints expressed by Welsh farmers, including environmental guardianship and interconnectedness of environmental, economic, social and cultural outputs, could assist in generating long-term positive outcomes (Davis, 2018).

## Conclusion

In this study, Welsh farmer perspectives on sustainable farming actions including barriers to implementation, decision-making processes, communication preferences, and suggestions for the future of sustainable farming were examined. Key barriers included time and cost to implement sustainable farming actions, availability of long-term financial valuation for ecosystem services, occurrence of extreme weather events, and presence of tenanted land (Table 2). Farmer-to-farmer knowledge transfer and decision-making through discussion with family, business partners, and other farmers aided participants in balancing perceived land hydrological needs with farm business requirements. Reflecting this community-based framework, participants highlighted interlinkage between environmental sustainability and broader aspects of social, economic, and cultural sustainability (Fig. 2). Design of tree-planting schemes was discussed as an example, with positive attitudes expressed towards countryside trees in wetter areas or on the margins of fields, but concerns raised towards farm-scale land sparing that reduced local housing and farmland availability (Fig. 3). This perspective unites environmental- and productivism-oriented motivations identified in a prior survey of Welsh beef and sheep farmer attitudes to climate change (Hyland et al., 2016).

In prior work, agrienvironmental subsidisation schemes have been linked to reduced economic barriers for to implementation of sustainable farming actions, increasing integration of environmental land management goals with farmer identities in England (Cusworth & Dodsworth, 2021). In a Bourdieuian framework, increased provision of economic, social, and cultural capital supported provision of symbolic (environmental) capital (Bourdieu, 1986; Cusworth & Dodsworth, 2021). Scheme design that incorporated farmer expertise in optimisation, maximisation, and efficiency by allowing for increased autonomy in design and implementation of sustainable farming actions was recommended (Cusworth & Dodsworth, 2021). These suggestions also address the time and cost barriers (Table 2) and decision-making processes that balanced environmental goals with farm business needs (Sect. 3.2) expressed by participants in this study. Participants described interconnected aspects of environmental, economic, social and cultural sustainability (Fig. 2), with similarities to Bourdieu’s theory. Welsh farmer participants in this study further identified the importance of a long-term outlook and interconnectedness between environmental, social, cultural and economic aspects (Fig. 2). Description of social and cultural aspects as related to the broader well-being of communities and longevity of Welsh language and culture emphasised these aspects. Initiatives that support the distinctive perspectives raised by study participants (Sect. 4.1, Fig. 2) could aid implementation of sustainable farming actions in Wales.

## Data Availability

The datasets generated during and/or analysed during the current study are available from the corresponding author on reasonable request.

## Abbreviation

NFU: National Farmers’ Union
